# Antitumor Activity and Potential Mechanism of Novel Fullerene Derivative Nanoparticles

**DOI:** 10.3390/molecules26113252

**Published:** 2021-05-28

**Authors:** Lianjie Ye, Larwubah Kollie, Xing Liu, Wei Guo, Xiangxian Ying, Jun Zhu, Shengjie Yang, Meilan Yu

**Affiliations:** 1 College of Life Science and Medicine, Zhejiang Sci-Tech University, Hangzhou 310018, China; 17865933628@163.com (L.Y.); larwubahkollie@gmail.com (L.K.); xingliu6699@163.com (X.L.); gw980310@163.com (W.G.); 2 Shaoxing Academy of Biomedicine, Zhejiang Sci-Tech University, Shaoxing 312030, China; yangshengjie@wahaha.com.cn; 3 College of Biological and Environmental Engineering, Zhejiang University of Technology, Hangzhou 310014, China; yingxx@zjut.edu.cn; 4 Hangzhou Wahaha Co., Ltd., Hangzhou 310018, China; zhujun13677@wahaha.com.cn

**Keywords:** fullerene, anti-tumor, cancer, nanoparticle, nanomedicine

## Abstract

The development of novel nanoparticles as a new generation therapeutic drug platform is an active field of chemistry and cancer research. In recent years, fullerene nanoparticles have received extensive attention due to their unique physical and chemical properties. Properly modified fullerene nanoparticles have excellent biocompatibility and significant anti-tumor activity, which makes them have broad application prospects in the field of cancer therapy. Therefore, understanding the anti-tumor mechanism of fullerene nanoparticles is of great significance for the design and development of anti-tumor drugs with low toxicity and high targeting. This review has focused on various anti-tumor mechanisms of fullerene derivatives and discusses their toxicity and their distribution in organisms. Finally, the review points out some urgent problems that need solution before fullerene derivatives as a new generation of anti-tumor nano-drug platform enter clinical research.

## 1. Introduction

The rapid development of nanotechnology provides new prospects for solving the existing limitations in biomedicine. The increased number of researchers dedicated to using nanomaterials and biotechnology and genetic engineering to diagnose, treat and prevent diseases. Fullerenes, since being discovered in 1985 by Kroto and Richard [1], have aroused widespread attention because of their excellent antioxidant properties. It is a kind of hollow molecule composed entirely of carbon and its shape is spherical, ellipsoidal, cylindrical or tubular. In addition, it contains not only six membered rings, but also five membered rings and occasionally seven membered rings. Fullerenes is an efficient “free radical sponge”, because it has obvious electrophilicity (the ability to accept up to six electrons). The solubility of pure fullerene in water is very low. In order to improve the biocompatibility of fullerenes, researchers made some modifications to fullerenes. Proper chemical modification of fullerenes dramatically improves their water solubility and biocompatibility, making these compounds extremely multi-use [2]. Table 1 summarizes the diversified mechanisms of fullerene family in cancer therapy.

Fullerenes can be modified by carbon cage internal nesting and carbon cage surface modification. On the one hand, the hollow interior of the carbon cage can contain single atoms, clusters of atoms and even small molecules [19,20]. Endohedral metallofullerenes (EMF) is formed by encapsulating various metal atoms or metal clusters in a fullerene cage [21,22]. Endohedral metallofullerenes has the comprehensive properties of carbon cage and internal metal part and has huge application potential as a molecular material. With the breakthrough of the large-scale production of metal fullerenes, the application research of metal fullerenes has made great progress and more and more new functions and practical applications have appeared [23]. Wang C et al. reported that RF-assisted gadofullerene nanoparticles induce rapid tumor vascular disruption by down-expression of tumor vascular endothelial cadherin [24]. Gadolinium metallofullerene (GMF) is a type of promising Magnetic resonance imaging (MRI) contrast agent with ultrahigh relaxivity [25]. Endohedral metallofullerenols [Gd@C_82_(OH)_22_]_n_ exhibit superb antineoplastic efficacy but low dose and toxicity, with no drug loading easily self-assemble to form relatively stable particles in many solvents, displaying strong antiproliferative effects on breast cancer cell line MCF-7, lung and hepatocarcinoma [26,27,28,29]. On the other hand, various polar groups (e.g., −OH, −COOH, −NH_2_) can also be attached to the surface of fullerene to improve its biocompatibility. Hydroxyfullerenes and other derivatized fullerenes are utilized due to their increased water solubility, thereby increasing the effective load for removing active oxygen from target cells and tissues. The proven protective effects of water-soluble fullerene derivatives include antioxidant and antimicrobial activities [30], scavenging free radicals [31] and anti-tumor activity [32]. Hydroxylated fullerenes and carboxyfullerenes are effective antioxidants and free radical scavengers, scavenging both the superoxide anion and H_2_O_2_, inhibiting lipid peroxidation and regulating apoptosis process in various biological model systems (e.g., cultured cells, mice and rats) [26,33]. Polyhydroxylated fullerenols regulate macrophages for cancer adoptive immunotherapy and greatly inhibit tumor metastasis, as reported [5]. Glycofullerenes as non-receptor tyrosine kinase inhibitors are studied towards better nanotherapeutics for pancreatic cancer treatment [34]. In general, the various derivatives of various fullerenes emergence makes the original fullerenes have more functions and further expands their application range from industry to medical field [35].

To date, the unique properties of fullerenes and their derivatives have attracted the researchers to develop high-efficiency antioxidants, solar energy converters, syntheticdiamonds, electronic materials, semiconductor devices and biomedicine. The research on the anti-tumor activity of fullerene occupies a special place in its possible application range. Compared with traditional antitumor drugs, fullerene derivative nanoparticles are non-toxic, it also has, including reversal of multidrug resistance (have no cross-resistance with traditional anticancer chemicals), immune activation, angiogenesis suppression, antioxidation, reduction of matrix metalloproteinase (MMPs) production and decrease in blood vessel density and nutrition supply to tumors versatile bioeffects [26,27]. In this review, we summarize the various unique antitumor activities of fullerene derivatives and systematically analyze their possible mechanisms from seven aspects, including cellular immunity activation, angiogenesis suppression, free radical scavenging, cell cycle regulation, tumor metastasis inhibition, photodynamic therapy and the antineoplastic drug delivery carriers.

## 2. Fullerene Derivatives: Promising New Anti-Tumor Nanoparticles

**Figure 1:**
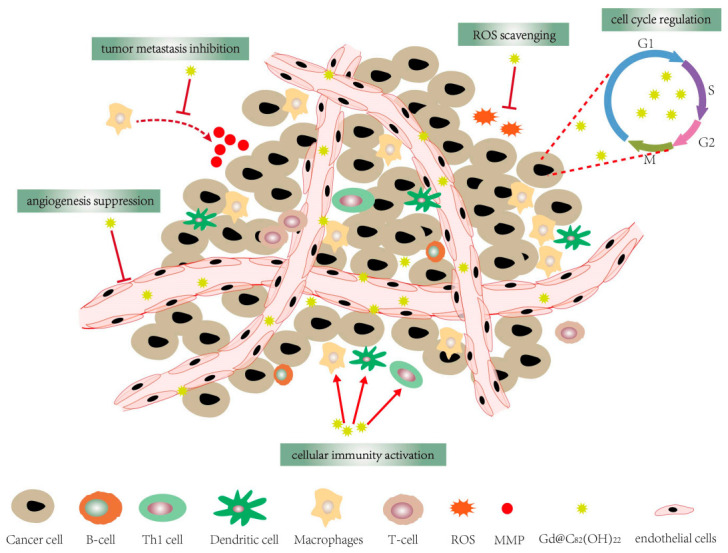
Gd@C_82_(OH)_22_ has a variety of anti-tumor activities, including cellular immune activation, angiogenesis suppression, cell cycle regulation, tumor metastasis inhibition and ROS scavenging.

There is growing evidence that fullerene-based nanomaterials, such as C_60_(OH)_n_, Gd@C_82_(OH)_22_, C_60_-paclitaxel conjugation and fullerenol conjugated with cisplatin (CDDP), etc. have different degrees of tumor-suppressing activity. C_60_(OH)_n_ (n = 6~24) has a stronger effect on inhibiting the proliferation of human breast cancer cells (e.g., T47D, MCF-7 cells and MDA-MB-231) with no visible damage to vital organs, compared with CDDP, doxorubicin, paclitaxel at equivalent concentration [26,27,36,37]. Growth-inhibitory effects of C_60_(OH)_n_/C_70_(OH)_n_ (n = 18–20) on human laryngeal cancer Hep-2 and cervical cancer HeLa cells are observed. In addition, C_60_-paclitaxel conjugation has produced tumor-suppressor activity on human epithelial lung adenocarcinoma A549 cells [38]. Fullerenol conjugated with CDDP shows significant inhibition of cervical cancer HeLa cells growth in vitro. Amphiphilic fullerene AF-1/paclitaxel nanocomposites result in a dramatic human MCF-7 tumor growth suppression with a similar inhibitory effect to Abraxane, a commercial nanoparticle of paclitaxel [39]. Stoichiometric pegylated DOX-C_60_ conjugates (1:1) and (2:1) with effective antiproliferative activity against MCF-7 cancer cell, which provides novel insights for nanomedicines incorporating tumor-targeting moieties and other [40]. CM9-fullerene complex showed a dose-dependent effect on survival of Jurkat leukemia cells in a dose-dependent way, presenting a potent candidate for acute leukemia treatment [41]. Likewise, [Gd@C_82_(OH)_22_]_n_ nanoparticles are reported to have high anti-tumor efficiency in a variety of cancer xenograft models such as liver cancer (H22, HepG2 cells) [26], human microvascular endothelial cells [42], MCF-7 cells [42,43,44]. In addition, Gd @ C_82_(OH)_22_ will not directly destroy the tumor, but by “blocking the cancer” to adjust the various components of the tumor microenvironment to exert its anti-tumor effect, which is different from the classic “kill cancer” therapy [14,26,45]. Even at very low doses, [Gd@C_82_(OH)_22_]_n_ can down-regulate the growth of transplanted liver cancer H22 cells and human breast tumors. Compared with common clinical anti-tumor drugs (such as CDDP and cyclophosphamide (CTX)), [Gd@C_82_(OH)_22_]_n_ can inhibit tumor metastasis without obvious side effects [14,26,36]. Furthermore, the growth rate of MCF-7 cells is cut by [Gd@C_82_(OH)_22_]_n_ with an average inhibition rate of 48%, whose antiproliferative activity is better than paclitaxel (45.1%) and CTX (41.0%) [26]. Meanwhile, Gd@C_82_(OH)_22_ inhibit the growth of Lewis lung cancer (LLC) and reduce the volume growth of human pancreatic tumors xenografted into mice [7]. Studies have shown that Gd@C_82_(OH)_22_ has a variety of anti-tumor activities (Figure 1), which is of great significance for multi-target therapy of tumors. Taken together, the excellent anti-tumor activity of fullerene derivatives and their unique interaction mode with specific targeting molecules may shed light on a new avenue for drug design. Therefore, understanding the potential anti-tumor mechanism of fullerene derivatives is of great significance to the design of nanomedicine. Now, we summarize the possible anti-tumor mechanisms of fullerene derivatives.

### 2.1. Cellular Immunity Activation

**Figure 2:**
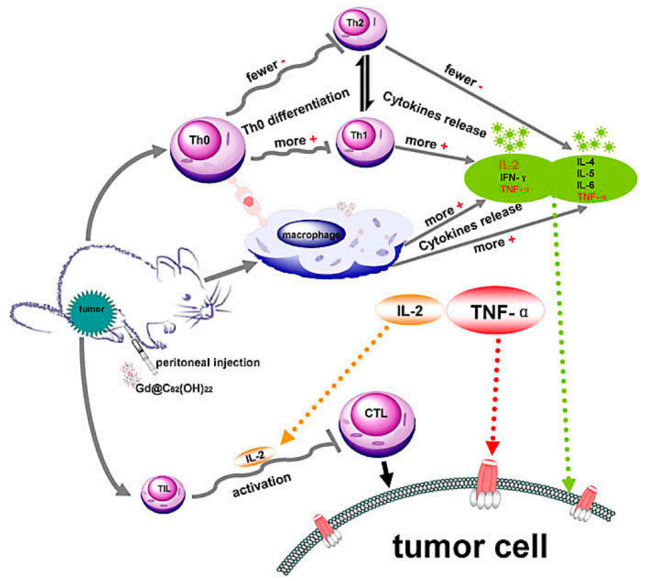
Possible immuno-mechanism related with the anti-tumor activity of Gd@C_82_(OH)_22_ particles [36].

Multipotent fullerene derivatives not only exert anticarcinogenic activity by regulating multiple phases in pivotal bioprocesses of cancer, i.e., carcinogenesis, tumor growth and invasion, but trigger a patient’s immune system to eliminate cancerous cells (e.g., melanoma, breast, prostate and leukemia carcinoma) [27,46,47,48]. It’s demonstrated that C_60_(OH)_20_ improve innate immunity by increasing CD_4_^+^/CD_8_^+^ lymphocyte ratio and enhancing TNF-α production to scavenge and prevent LLC cells and its efficient antitumor activity against LLC cells is similar to Gd@C_82_(OH)_22_ [3]. [Gd@C_82_(OH)_22_]_n_ induce dendritic cells (DCs) to become functionally mature and activate allogeneic T cells in tumor-bearing mice to eradicate foreign pathogens and scavenge tumors and depress the viability of the tumor by activating TNF-α mediated cellular immunity (Figure 2) [6,36,46,49,50]. These nanoparticles can effectively improve the immune system including polarizing the cytokine balance towards Th1 (TNF-α, IFN-γ, IL-2) cytokines and lowering the production of Th2 (IL-4, IL-5, IL-6) cytokines [6,36]. Gd@C_82_(OH)_22_ strongly activates primary mouse macrophages and produces pro-inflammatory cytokines to promote tumor immune-inhibition and not interfere with either the viability or growth of human primary immunocyte (B cells, T cells, or macrophages), nor induces the cell apoptosis, evidencing their possible role in antitumor immune response [5]. Moreover, Gd@C_82_(OH)_22_ greatly stimulates IL-1β secretion of macrophage via both TLRs/MyD88/NF-κB pathway and NLRP3 inflammasome activation, while Gd@C_82_(OH)_22_ shows better ability than C_60_(OH)_22_ for electron affinity on the surface of carbon cage induced by encaged gadolinium ion [46]. However, both C_60_(OH)_20_ and Gd@C_82_(OH)_22_ could release several lines of cytokines, such as IL-2, IL-4, IL-5, TNF-α and IFN-γ [36], inhibiting the tumor growth in vivo. C_60_ fullerene derivatives conjugated with bovine thyroglobulin or rabbit serum albumin induce the generation of fullerene-specific IgG antibodies in immunized mice [51]. Bis-malonic acid fullerene derivative compound inhibits interleukin 33 (IL-33)-induced expression of IL-6 in bone marrow-derived mast cells (BMMC) [52]. Recently, Wang C et al. reported that gadofullerene (GF-Ala) nanoparticles are demonstrated to reprogram TAMs to M1-like and increase the infiltration of cytotoxic T lymphocytes (CTLs), achieving effective inhibition of tumor growth [53]. In general, fullerene derivatives provide a new path for targeted therapy of tumors by activating host immunity.

### 2.2. Angiogenesis Suppression

The growth of tumors depends on tumor angiogenesis and adequate blood supply. Therefore, anti-angiogenesis is an effective strategy for cancer treatment. The inhibitory effects of fullerene derivatives on carcinoma growth and metastasis in vivo are involved in oxidative modulation and angiogenesis. Oxidative stress in carcinoma cells promotes blood supply to tumors by increasing angiogenic factors interleukin-8 (IL-8), vascular endothelial growth factor (VEGF) and MMPs secretion of endothelial cell and many other ways to play their collaboration effect. These particles prevent cancer by inhibiting oxidative stress, further reducing tumor vascularization [8]. Specific blockage of tumor angiogenesis provides a new idea for the clinical treatment of drug-resistant solid tumors. Gd@C_82_(OH)_22_, a potent angiogenesis inhibitor, significantly reduces tumor microvessels density (MVD) more than 40%, lowers the speed of blood supply to cancer tissues by 40% and can simultaneously down-regulate the expression of more than 10 angiogenic factors at mRNA and protein level [7,13]. For in vivo malignant human breast cancer models, the antitumor activity of Gd@C_82_(OH)_22_ is essentially associated with the reduction of several pro-angiogenic factors like MMPs, thereby suppressing neovascularization and inducing anti-metastatic imprisonment of cancer tissue [13]. In addition, Gd@C_82_(OH)_22_ specifically targets intrinsic cancer stem cells (CSCs), which represents a novel approach to suppress tumor angiogenesis without exacerbating the crucial cancer cell population [26]. In addition, fullerenols decreases intra-tumor MVD in vivo, probably by down-regulating TNF-α, VEGF and PDGF expression [8,9]. Similarly, C_60_(OH)_20_ reduces oxidative stress and generation of angiogenic factors and neovascularization within breast carcinomas, possibly through a decrease in the expression of TNF-α, VEGF and PDGF [4]. Fullerenol–Dox conjugates with 16–24 hydroxyl groups or poly(ethyleneglycol)(PEG) activate excellent antiangiogenic activity in murine tumor angiogenesis models, which is rapidly internalized into endolysosomal compartment [54].

### 2.3. Reactive Oxygen Species (ROS) Scavenger of Fullerene Derivatives

The balance of free radicals in the body plays an essential role on keeping healthy. There is increasing evidence that excess free radicals can cause oxidative stress and metabolic disorders, leading to various human diseases [55,56,57]. Fullerene, known as a “radical sponge” [58], can react readily with free radicals like a detoxifying system to effectively quench active radicals or suppress ROS to lower free-radical-induced detrimental effects and maintain ROS in biological systems [59]. The protective activity of water-soluble nano-C_60_ derivatives and Gd@C_82_(OH)_22_ to impede the chain reaction of lipid peroxidation could be partly attributed to their free radical scavenger properties [33]. It has been reported that fullerenols can effectively absorb superoxide (O_2_^−^) generated by xanthin/xanthine oxidase, diminish hydroxyl radicals in human breast carcinoma cell lines, reduce lipid peroxidation and restore glutathione (GSH) in the small intestine after I/R injury in dogs [30,60]. C_60_(OH)_n_ have high radical-scavenging and neuroprotective activity relying on the effect of dose and time, it can stimulate the autophagic response in tumorigenesis, metastasis and induce cell apoptosis [61,62]. Its radioprotective effects are concentration-dependent. Meanwhile, its main mechanism of protecting mitochondrial function is to prevent protein damage through anti-oxidation, maintain mitochondrial membrane potential and induce apoptosis [63]. C_60_(OH)_24_ attenuates H_2_O_2_-induced apoptotic cell death in A549 cells via a fortifying Nrf2-regulated cellular antioxidant capacity [14]. C_60_(OH)_24_ has strong antioxidant properties by influencing the cellular redox state and enzyme activities to reduce cell proliferation [64]. In addition, fullerenol nanoparticles protect from doxorubicin-treated hepatopathy [65,66]. C_60_(OH)_n_ (n = 22–24) could also protect rat hepatocytes in a dose-dependent manner against carbon tetrachloride (CCl_4_) acute toxicities by scavenging large amounts of radicals. Likewise, C_60_(OH)_7±2_ reduces H_2_O_2_-induced ROS formation and protects against oxidative stress in RAW 264.7 cells and ischemia-reperfused lungs. C_60_(OH)_24_ has a protective effect on the lungs and prevent doxorubicin-induced lung toxicity through inhibition of oxidative stress in tumor-bearing rats after treatment with a single dose of doxorubicin, which is under the previously reported sparing effects of nanoparticles on inhibition of doxorubicin-mediated toxicity in lungs, kidney and testes of Wistar rats. However, water-soluble C_60_ derivatives prevent NO-mediated cell death in rat pheochromocytoma cells without clear toxicity [67]. Highly hydroxyl-functionalized C_60_(OH_)36_ · 8H_2_O protects cells from H_2_O_2_-induced oxidative injury by scavenging hydroxyl radicals (∙OH) and oxidizing them to a potentially stable dehydrogenated fullerenol radical C_60_-O∙ [68].

Furthermore, Gd@C_82_(OH)_22_ possesses the powerful antioxidative ability, e.g., decomposing ROS (such as O_2_•, HO•, ^1^O_2_), eliminate stable DPPH•free radicals and effectively inhibiting lipid peroxidation in vitro, thus protecting normal cells and their sub-cellular environments against oxidative injure [26]. In A549 cells or rat brain capillary endothelial cells(rBCECs), [Gd@C_82_(OH)_22_]_n_ has a strong inhibitory effect on H_2_O_2_-induced ROS formation and mitochondrial damage [69]. [Gd@C_82_(OH)_22_]_n_ restores hepatic and renal functions in vivo and regulates oxidative stress in malignant cells. These Nanoparticles (NPs) mitigated damage to the main organs such as liver and kidney of tumor-bearing mice by minimizing the metalloprotease enzyme activities while restoring other parameters after Gd@C_82_(OH)_22_ treatment in vivo and/or in vitro, such as oxidative stress, close to normal levels [10]. Gd@C_82_-(EDA)_8_ NPs with high cellular uptake and ROS scavenging ability, display an outstanding cytoprotective effect against H_2_O_2_-induced injuries to human epidermal keratinocytes-adult (HEK-a) cells even at a very low concentration of 2.5 μM, compared with that of Gd@C_82_(OH)_26_ at a much higher concentration of 40 μM [15]. Comparatively, C_60_(C(COOH)_2_)_2_, C_60_(OH)_22_ and Gd@C_82_(OH)_22_ eliminate physiological ROS and protect A549 and rat brain cells (rBCEC) against oxidative damage, stabilize MMP and attenuate intracellular ROS formation with the following relative potencies: Gd@C_82_(OH)_22_ > C_60_(OH)_22_C_60_(C(COOH)_2_)_2_ [70]. Indeed, if a nanomaterial can effectively reduce ROS level in neoplastic cells, it has a certain anti-cancer property in theory. Using fullerene derivatives as anticarcinogenic nanodrugs to rationally regulate ROS level, enhance carcinoma inhibition and protect normal tissues from tumor invasion.

### 2.4. Cell Cycle Regulation

A therapeutic strategy for inducing DNA damage in tumors might be promising for killing cancer. C_60_(OH)_16–24_/DOX nanocomposites in vitro decrease the growth of mouse melanoma cell line (B16-F10), mouse lung carcinoma (LLC1) and human MDA-MB-231) through an arrest of G2-M cell cycle inducing an increase in DNA damage. In an in vivo murine tumor model, the FNP/DOX conjugates significantly suppress the growth of MCF-7 at all examined doses and MDA-MB-231 cells at lower doses by blocking G2-M progression and triggering DNA damage, thus resulting in apoptosis, which gives comparable anticancer efficiency as free DOX with no typical adverse effects [37,71]. One of the possible mechanisms by which C_60_(C(COOH)_2_)_2_ induce cytotoxicity, is through cell cycle block, causing cell apoptosis. The growth of tumor cell could also be inhibited by [Gd@C_82_(OH)_22_]_n_ NPs by regulating the cell cycle,, which may explain the low toxicity of nanoparticles. Zhao Y et al. reported that [Gd@C_82_(OH)_22_]_n_ inhibits both MCF-7 and ECV304 cell growth by inducing the G0/G1 phase arrest [11,12]. [Gd@C_82_(OH)_22_]_n_ naturally prevent the entry of cells into the S phase and induce cell apoptosis by down-regulated expression of CDK4, CDK6, cyclinE, cyclinD2 and Bcl-2 and by up-regulated expression of P21 and Bax [12]. A fullerene nanoconjugate with gemcitabine can inhibit the S-phase cell cycle and induce apoptosis [72]. Based on all the aforementioned ideas, a nanomedicine platform affecting cell cycle distribution can be used as a pharmacon to cure tumors.

### 2.5. Tumor Metastasis Inhibition

**Figure 3:**
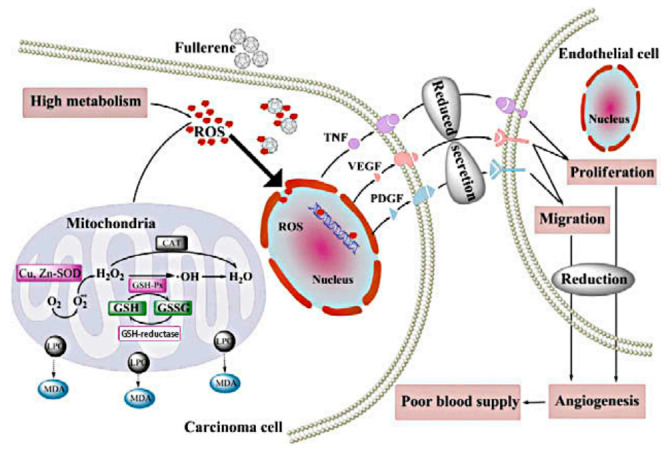
Possible anti-metastatic mechanism of C_60_(OH)_20_ nanoparticles in vivo [4].

**Figure 4:**
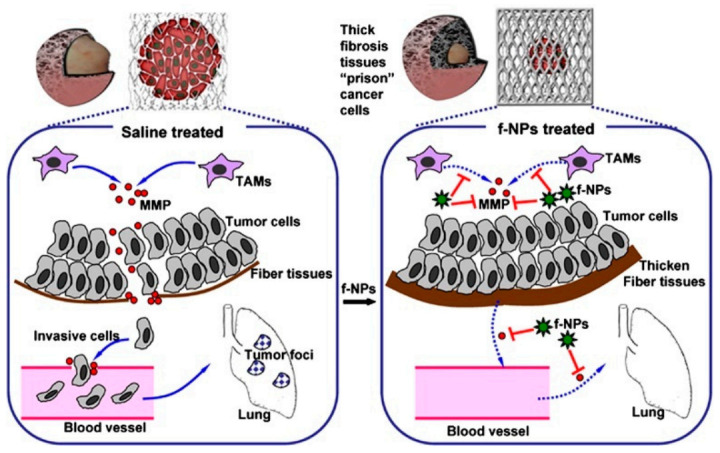
Mechanism by which Gd@C_82_(OH)_22_ block carcinoma metastasis mainly via an MMP-inhibition process. The thick fibrous cage might serve as a ‘prison’ to tightly confine invasive tumor cells within the primary siteb [13].

**Figure 5:**
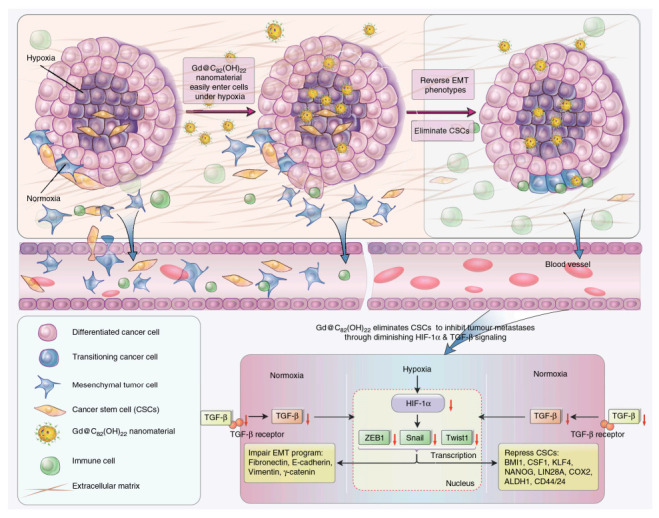
Key pathways by which Gd@C_82_(OH)_22_ NPs suppresses tumor growth [80].

Metastasis remains a tremendous hurdle and a major contributor to the majority of cancer deaths. Related articles showed that some fullerene derivatives suppress cancer metastasis effectively (Figure 3 and Figure 4) [4,13]. C_60_(OH)_20_ aggregates possess the anti-metastatic activity in EMT-6 breast carcinoma models. More specifically, matrix metalloproteinases (MMPs) by extracellular matrix (ECM) degradation and mediating ECM-bound pro-angiogenic factors like VEFG and FGF-2 release, play a crucial role in tumor proliferation and migration, making them potential targets of anti-metastatic nanotherapeutics [73,74]. Down-regulation of MMPs expression in the tumor microenvironment might significantly reduce the metastatic potential of carcinoma [42]. Intriguingly, Gd@C_82_(OH)_x_ (x = 20–24) effectively restricts carcinoma metastasis and invasion mainly by an MMP-suppressive process by prevention of MMP expression (MMP-2, especially MMP-9) and angiogenesis factors inhibition, which imprison rather than poison cancerous cells, with no apparent toxic effects on normal cells [6,7,27,31]. Small size fullerenol nanoparticles suppress lung metastasis of breast cancer cells by disrupting actin dynamics [75]. A thick fibrous layer in the extracellular matrix through regulating the TNF-α signaling pathway in CAFs and fibrosarcoma cells formation could be contributed by Gd@C_82_(OH)_22_, thereby blocking tumors and inhibiting tumor metastasis [76]. In addition, Gd@C_82_(OH)_22_ affects the epigenetic regulation of human pancreatic cancer metastasis and suppresses pancreatic cancer cell invasion and metastasis by down-regulating metastasis-related protein 1 (MTA1), HDAC-1, HIF-1R and MMP-2/9 and up-regulating reversion-cysteine protein with the Kazal motif (RECK) expression [77,78]. In polyoma middle T–induced mammary cancer mouse models, lung metastases is decreased by the depletion of macrophages [79]. C_60_(OH)_22_ or Gd@C_82_(OH)_22_ regulate macrophage for cancer adoptive immunotherapy, inhibit cancer cell growth through NF-κB-mediated release of multiple cytokines and greatly suppress cancer cell metastasis to the lung. Gd@C_82_(OH)_22_ shows stronger immuno-modulatory effects on macrophages function than C_60_(OH)_22_ [5]. More interestingly, Gd@C_82_(OH)_22_ prevents epithelial-to-mesenchymal transition, suggesting that triple-negative breast CSCs can be effectively eliminated in vivo and in vitro and tumorigenesis and metastasis of cancer cells can be inhibited by this intervention, indicating that non-toxic Gd@C_82_(OH)_22_ selectively targets CSC under hypoxic conditions without targeting normal or normal stem cells (Figure 5) [80].

### 2.6. Fullerene Derivatives for Tumor Photodynamic Therapy: DNA Cleavage and Membrane Damage

Photodynamic therapy (PDT) employs the combination of non-toxic photosensitizers (PS) and harmless visible light to a tumor region at the surface of the body or to internal tumors to generate cytotoxic ROS for killing cells. Compared with other treatment methods, PDT has many advantages. However, under the hypoxic environment of tumors, PSs40 has poor skin sensitivity, poor tumor targeting and poor therapeutic effects, which limits the application of PDT [81]. Functionalized or solubilized fullerene derivatives are a novel PS capable of absorbing visible light and then generating ROS through an electron transport chain and induce severe adverse effects to intracellular critical biomolecules (nucleic acids, lipids, proteins) to cause cancer cell apoptotic or necrotic death, without any damage to normal skin [82]. On the one hand, photoactivated DNA cleavage by nanoparticles under visible light irradiation offers new therapeutic strategies for tumors. A fullerene carboxylic acid produces cleavage of supercoiled pBR322 DNA strands after illumination. In presence of NADH, fullerene conjugated with an acridine molecule as a DNA intercalator, whose DNA photo-cleavage activity on pBR322 supercoiled plasmid is much more effective than pristine fullerene C_60_ under irradiation [83]. DNA cleavage activity of a β-cyclodextrin fullerene C_60_ compound can be seen by using supercoiled pBR322 plasmid DNA under visible light irradiation. In addition, the compound shows no significant cytotoxicity in human neuroblastoma SH-SY5Y cells [84]. Fullerene-oligonucleotide binds to certain single or double-stranded DNA and cleaves the strand(s) proximal to the fullerene moiety under the light. DNA photocleavage capacity of fullerene oligomer and functionalized C_60_ has also been reported briefly. Water-soluble anthryl-cyclodextrin + C_60_ conjugate has cleavage efficiency in pGEX5X2 DNA [85]. Functionalized liposomes incorporating both C_60_ and C_70_ into the lipid bilayer can photocleave ColE1 supercoiled plasmid DNA upon exposure to λ > 350 nm light. Interestingly, C_70_ is more effective (3.5 times) than C_60_ in DNA cleavage. On the other hand, Fullerenes derivatives are capable of triggering ROS production upon illumination with UV and visible light and initiating lipid peroxidation and protein oxidation in membranes. Experiments in vitro indicate that C_60_ derivatives cause HeLa cell membrane damage by light irradiation and accelerate the irreversible cell death. C_60_(OH)_18_ induces cell membrane injury through time-and-dose dependent oxidative damage after photoexcitation in rat liver microsomes model [86]. Significant damage induced by pristine C_60_ is largely due to ^1^O_2_, whereas that by C_60_(OH)_18_ is mainly because of reactive species. They differ somewhat in damage for both lipid and protein and the latter is more pronounced. A water-soluble pristine C_60_ fullerene suspension generates ROS under photoactivation and induces cell membrane damage, however, DNA concentration and mitochondrial activity are less uninfluenced [87].

Moreover, the photodynamic action of these nanostructures against malignant cancers (e.g., lung, cervical and colon carcinoma) in vivo, is a negative correlation between the extent of C_60_ derivatization and a corresponding reduction of C_60_ ROS-generating ability. Three C_60_ derivatives with two to four malonic acid groups (dimalonic acid C_60_ (DMAC60); trimalonic acid (TMAC_60_); and tetra-malonic acid C_60_ (QMAC60)), whose phototoxicity to HeLa cells is as follows: DMAC60 > TMAC60 > QMAC60. Fullerene derivatives containing greater ^1^O_2-_quenching groups (–OH) show lower photodynamic activity, as opposed to those containing equal numbers of groups (–CH) with lower ^1^O_2_-quenching effects [88]. In addition, C_60_-PEG conjugate, a good photodynamic therapeutic for tumor, exhibit more favorable accumulation in neoplastic tissue and induce tumor necrosis with non-toxicity to normal skin [26]. Furthermore, after intravenous injection of C_60_-PEG conjugate with Photofrin^®^, a commercially available PDT agent, into tumor-bearing mice, tumor volume is inhibited upon exposure of tumor site to visible light in a light-dose-dependent manner. Pullulan-modified fullerenes show superior photo-physical qualities and ROS scavenging when irradiated with visible light, not only as antimicrobial photosensitizers but drugs for photodynamic diagnosis and therapy of tumors [89]. Pyrrolidinium-C_60_ derivative is a highly efficient PS and can kill three mouse cancer cell lines of colon adenocarcinoma (CT26), J774 reticulum sarcoma and LLC even at low concentration by exposure to white light. In addition, nanoparticles induce cell apoptosis in CT26 cells after illumination and phototoxicity might be mediated by ^1^O_2_ and superoxide [89]. ROS production in mitochondria plays a crucial role in PDT. At low doses of chitosan oligosaccharide grafted fullerene conjugate (CS-C_60_) compound, ROS generation is concentrated in mitochondria and enhances the suppression of human malignant melanoma (A375) cells [90]. C_60_-mediated PDT is a good candidate for restoring drug-resistant leukemic L1210 cell lines induced to mitochondrial apoptosis [91]. Some recent studies have shown that the rational modification of fullerene nanoparticles can definitely enhance the effect of photodynamic therapy. Fullerene-nanodiamond composite is internalized by cancer cells and induced cell apoptosis without noticeable toxicity [92]. NIR light-harvesting fullerene-based nanoparticles (DAF NPs) can effectively inhibit tumor growth through synergetic photodynamic and photothermal therapies, which provides a new sight of photosensitizer design for enhanced cancer phototheranostics [93]. A C_60_ based hybrid nanostructured photosensitizer (C_60_@GNPs), taking advantage of the surface plasmon resonance (SPR) effect of gold nanoparticles (GNPs) and the “electronic sponge” property of C_60_, can effectively produce hydroxyl radicals under the excitation of near-infrared light to kill tumor cells [94]. Recently, Wang C et al. reported that the high yields of photo-induced ^1^O_2_ by β-alanine modified gadofullerene nanoparticles (GFNPs) can lead to the destruction of the tumor vascular endothelial adherent junction protein-VE cadherin and the decrease of tumor vascular endothelial cells-CD31 proteins, inducing the rapid tumor necrosis [16]. Thus, fullerene derivatives are promising photodynamic therapeutic systems for carcinoma diagnosis and therapy, though several years are in demand for clinical trials.

### 2.7. Fullerene Derivatives: The Antineoplastic Drug Delivery Carriers

Nanomaterials vectors, with small size and large surface area, unmatched flexibility and diversity to endow polymers, liposomes, inorganic materials and carbon allotropes with promising in drug delivery systems, may favor passive targeting drug molecule into body sites with leaky vasculatures, such as tumors and inflammation sites. Moreover, nanoparticles-based nanocarrier, a new kind of carbon vector for existing therapeutic agents, oligonucleotides or bioactive molecules, will accelerate the drug into the cells and enhance drug targeting, lessening their toxic side effects and/or improving their pharmacokinetic properties in cancer ablation [95,96,97].

Since water-soluble fullerene derivatives can effectively carry polar drug molecules such as DOX and paclitaxel to enter tumor cells, even into the deep tissue. Therefore, it could become an ideal vehicle for antitumor drugs and it might be helpful if fullerene derivatives are possibly guided by targeting molecules to improve curative effect and cut many side effects [39,71]. For example, C_60_(OH)_n_ (n = 2–26) work as a carrier for cancer drugs buckysomes [54,71]. The therapeutic potential for a fullerene-based amino acid depend on its use as a carrier for the transport of nuclear localization sequence peptide, which can greatly be adsorbed to around the nucleus of neuroblastoma and HEK-293 cells [98]. There is increasing evidence that fullerenes can possibly be used as anti-tumor drug carriers with traditional anti-tumor drugs to improve anti-tumor effects and reduce toxic side effects. C_60_ fullerene-based delivery nanocomplexes had a potential value for optimization of doxorubicin efficiency against leukemic cells [99]. C_60_ fullerene, as a component of the C_60_–Cis-Pt nanocomplex has been reported to increase the Cis-Pt entry and an intracellular accumulation thus, contributing to the drug’s toxic effect [17]. Fullerenol/Dox nanocomplexes, with a diameter of 50–80 nm and about 25% drug loading efficiency, significantly reduce the growth of melanoma and LLC cells and induce cell apoptosis in vitro and prohibit B16/F10 tumor growth in vivo [71]. In addition, conjugation of Dox to fullerenols increases its therapeutic index and decreases its system toxicity. In addition, research has shown that the complexation of Alkaloid Berberine with C_60_ Fullerene, as a nanocarrier, enhanced its uptake by leukemic cells with toxic effects, which provided a proof of concept of the strategy of using C_60_ for natural medicine nanodelivery [18]. Many hydroxyl groups in the surface of fullerene molecule could be the passport to sparingly water-soluble or hydrophobic cytotoxic drugs and provide a route for multiple hydrogen bonds with various components, ameliorating hydrophilicity and intracellular delivery of drug molecules. Moreover, the stratagem for a drug delivery system tailored is to identify specific targets (especially those over-expressed in carcinoma) by tagging nanoparticles with small molecules, oligonucleotides, or antibodies. The process of targeted nanodrugs delivery can be guided with passive or active targeting. Under passive targeting, drug loading behavior is elevated, provided that, due to the enhanced permeability and retention (EPR) effect; by which nanodrugs potentially cross irregularly vascularized (“leaky”) veins in tumor tissues and accumulate preferentially at a cancerous site showing lowered lymphatic drainage activity. However, active targeting mostly depends on the diffusion through the blood cycle system and specific interaction between target cells (or target intracellular components). Nanoparticles are likely responsible for active targeting, but the evidence supporting this position is not compelling. The comprehensive judgement of nanoparticles-based drug delivery system may involve cytotoxicity of nanocomposite itself and their potential to induce DNA damage. DOX-loaded FNP as a potential intracellular drug-targeting delivery system raises cellular DOX uptake, exhibits significant cytotoxicity effects and reduces MCF-7 and MDA-MB-231 cell growth, inducing an increase in DNA damage. However, this conjugate is not well-defined structurally, which presents a serious obstacle concerning its applicability in clinical practice. In general, future trends point toward more efficient nanodrugs and nanocarriers, as well as “nanodevices” that integrate both nanocarriers and nanodrugs, which provide an alternative and novel approach for overcoming some of the difficulties experienced by the traditional modality of delivering small molecule-based drugs.

## 3. The Advantages of Fullerene as a Potential Anti-Tumor Nano-Drug

Clinical treatment methods for tumors mainly include surgery, chemotherapy and radiotherapy. Chemotherapy occupies a very important position in cancer treatment, but chemotherapy drugs have problems such as poor solubility, poor biocompatibility and poor selectivity, which will cause many toxic side effects and ultimately may lead to chemotherapy failure [100]. Fullerene derivatives not only have a variety of anti-tumor activities, but also have characteristics that traditional drugs do not have, such as large specific surface area, highly specialized nanostructures and electron affinity caused by surface chemistry. Therefore, compared with traditional anti-tumor drugs, fullerene derivatives have the characteristics of good water solubility, low toxicity and good tumor targeting.

In detail, due to multi-polar functional groups (e.g., −OH, −COOH, −NH_2_) overcoming fullerenes’ insolubility in water and decreasing their cytotoxicity, while retaining their unique properties and enhancing their biological activities, fullerene derivatives can not only target, image the existence of neoplasia and detect pathophysiological defects within the tumor, but also physiologically respond to an external stimulus to control the release of drug and recognize residual tumors [101]. Some fullerene derivatives with supported targets locate a few major pathways, allowing certain tumors to successfully shrink. Solubility enhancement of fullerenes in water/biological media increases the drug concentration at the desired site and promotes optimal targeted therapy for solid tumors. Furthermore, fullerenes structural conjugate drugs (e.g., PEG, surfactants, calixarenes and cyclodextrins) attachment occurred by bio-stability and convenient three-dimensional scaffolding. Those hydrophilic groups or ionic species confer surfactant-coated C_60_ and Gd@C_82_(OH)_n_ with manifold antineoplastic effects including drug and gene delivery, DNA photocleaving, lipid peroxidation inhibitor, antioxidation, anti-metastasis, anti-apoptosis and immunomodulatory activity [26,102]. In addition, with a large surface-to-volume ratio allowing multiple functional groups or “blocks” to be attached to fullerenes’ surfaces, their derivatives can cross the physiological barrier in vivo quite easily and increase drug distribution and targeting, reducing negative effects often presented in traditional drugs like low target selectivity, poor release control and easy resistance [27,102]. Gd@C_82_(OH)_x_, a new type of nanoparticles with a large surface-to-volume ratio, is reported to efficiently shocks a wide array of interactions with proangiogenic factors like MMPs and their mRNA precursors and blocks loosely modulatory, chaotic blood vessels in tumor tissues. Meanwhile, fullerene derivatives with highly specialized nanostructure, electronegativity and nanoscale, make them effective tools for antioxidation and drive their role in ROS scavengers. Physiochemical property (i.e., differences in electron affinity and aggregation state induced by surface chemistry) may influence its biological and biomedical effects drastically [6]. The size and morphology of C_60_ derivatives also affect the ROS quenching activity [103]. Furthermore, the alterations of biomechanical properties, particularly, particle adhesion and small size which are close linkages with cellular migration, growth and differentiation, are beneficial for fullerene derivatives to improve the retention of local medication, increase contact time and contact area, enhancing immunity and protecting normal tissues from tumor invasion. The dynamic adhesion characteristics of C_60_(OH)_24_ treated human hepatoma cells (SMCC-7721) help to reveal the biomechanical behavior of cells. In addition, a negative charge on the surface makes nanoparticles easier to remove in vivo, whereas a neutral charge induces prolonged cycle time in the body. Identification of specific biological processes and biochemical pathways in cells treated with nanoparticles may profit to develop the potential bioeffects and biosafe anticarcinogen in vitro and in vivo.

## 4. Toxicity and Biodistribution of Fullerene Derivatives

The biodistribution and toxicity of nanomaterials must be considered before clinical application. Due to the difference in the number of carbon atoms of fullerenes (for example, C_60_, C_70_, C_80_ and C_94_) and differences in the preparation process of fullerenes, the physical and chemical properties of fullerene derivatives show great differences. Meanwhile, the alterations in physicochemical properties imposed by the utilization of different methods for C_60_ solubilization profoundly influence toxicological effects of fullerene preparations, thus making the analysis of their potential therapeutic and environmental toxicity difficult. The “cytotoxicity” of fullerene derivatives (either covalently or noncovalently) promotes their utilities in fighting against tumors by “poisoning” cancer cells. New evidence demonstrates that in vivo fullerenols (e.g., C_60_(OH)_7±2_ and C_60_(OH)_22–26_) elicit toxious to different cell lines such as mouse osteogenic sarcoma, human vascular endothelial, lens epithelial, RAW264.7, HeLa cells, among other things [49]. Furthermore, fullerenols exert certain cytotoxic/phototoxic, genotoxic and mutagenic effects on CHO, HaCaT, Hela and HEK293 cell lines, respectively [104]. Toxic effects of fullerenoles may be caused by mitochondrial dysfunctions associated with induction of membrane permeability transition (MPT), depolarization of inner mitochondrial membrane, inhibition of oxidative phosphorylation in the early stage and subsequently reduction of cellular oxidation, GSH, protein thiols, malondialdehyde (MDA) formation and lipid peroxidation at a later stage [104,105]. Potential toxin of C_60_(OH)_44_ to isolated mitochondria not only affect the respiratory chain but damage the inner membrane, allowing permeability to H+ and K+, which elucidates unknown function at subcellular level [106]. Additionally, pyrrolidinium fullerene is exemplified for affecting tumor cell growth and inducing apoptotic cell death of cells mutated on JAK2 V617F by depletion of apoptosis signal-regulating kinase 1 (ASK1) and inactivation of c-Jun N-terminal kinase (JNK) pathway, which illustrates resistance to multiple anti-tumor drugs [107]. Pyrrolidinium fullerene modified with a suitable length of alkyl group, increases its apoptotic effect by resistance of the ASK1-MKK4/7-JNK pathway and decreases cytotoxicity mediated by ROS production to human promyeloleukemia (HL-60) cells [108]. In addition, replacement of the N-methyl group or one hydrogen atom on compound with a butyl group can enhance the activity of pyrrolidinium fullerenes in inducing VF-Ba/F3 cells apoptosis [107]. High dose of fullerene derivatives C_60_-(OH)_20_(HFD), C_60_-(Arg)_8.6_(RFD), C_60_-(Lys)_8.7_(KFD), C_60_-(NH(CH_22_)_2_NH_2_)_8.8_(NFD) and C_60_-(β-Ala)_10.1_(AFD) could induce growth inhibition and toxicity of cells in the RAW264.7 through the apoptosis pathway. Moreover, autophagy and lysosomal dysfunction caused by nanomaterials result in emerging toxicological consequences [43]. Interestingly, there are also some reports showing that the toxicity of water-soluble carboxylated C_60_ and C_60_(OH)_n_ (n = 2–72) was not found in vitro, in vivo and clinical reports. C_60_(OH)_20_ shows nearly no toxic effects on human breast cancer MCF-7 cells [44]. In addition, the potential tumoricidal activity of [Gd@C_82_(OH)_22_]_n_ including the regulation of oxidative ability, the improvement of cell-mediated immunity and the angiogenesis inhibition is superior to C_60_ fullerenols [27,46]. No abnormal pathological changes are found in normal mammary epithelial cells when treated with Gd@C_82_(OH)_22_ [45]. Moreover, water-soluble fullerene derivatives show no acute toxicity after oral administration in rats and have different affinities in vivo because of their diversity of water solubilizing groups. Increased studies have shown that the toxicity of fullerenes and their derivatives may be related to the preparation process, purity and concentration of fullerenes. In particular, trace impurities in fullerenes may be an important factor leading to the toxicity of fullerenes. Of course, more experimental studies are needed to evaluate the toxicity of fullerenes and their derivatives.

Lines of evidence have identified the distribution and accumulative pattern of nanopharmaceuticals in drug-resistant tumor models. [Gd@C_82_(OH)_22_]_n_ interfere with tumor invasion in normal muscle cells and are only less than 0.05% of the injected dose within carcinoma tissues [26]. These nanoparticles are mainly accumulated in the bone (52.45 ng Gd/g wet weight), pancreas (20.33 ng Gd/g wet weight), kidney (12.73 ng Gd/g wet weight), liver (11.56 ng Gd/g wet weight) and spleen (7.52 ng Gd/g wet weight) via intraperitoneally (ip); only a small quantity distribute in the lung (3.41 ng Gd/g wet weight) and tumor tissues (3.76 ng Gd/g wet weight), but could hardly penetrate the blood-brain barrier [26]. [Gd@C_82_(OH)_22_]_n_ are less concentrated in serum (1.43 ng Gd/mL) and red cells (0.4 ng Gd/g wet weight). Around about 50% of Gd@C_82_(OH)_22_ are evacuated from urine and 35% from feces. The results indicated that these particles reach the organs and tissues through vascular circulation and cannot remain in the blood at 24 h after administration, similarly with the biodistribution of 166Hox@C_82_(OH)_y_ in BALB/c mice after 24 and 48 h of injection [26,27]. Approximate 20% of C_60_(OH)_24_ exists in the abdomen, especially on the ventral surface of the liver, spleen and pancreas and in the ventral mesenteric fat area in the perihepatic region of the liver [65]. 99mTc-C_60_(OH)_x_ accumulates in bone, kidneys, spleen and liver via penetrating reticuloendothelial cells [109]. 90–95% of ^14^C–labeled pyrrolidine fullerene particles are distributed over various organs after intravenous injection to SD rats, which mainly enrich into the liver, sparingly penetrate the blood-brain barrier and are rapidly through the renal excretion. Carboxylic acid fullerene C_60_C(COOH)_2_ is mainly located in mitochondria and cell membrane; C_60_(C(COOH)_2_)_2_ is concentrated in cellular lysosomal, not in the mitochondria; C_60_(C(COOH)_2_)_3_ is partly positioned in cellular mitochondria.

## 5. Conclusions and Perspectives

Fullerene derivatives have comprehensive properties of fullerenes and modified groups and have great application prospects in the field of medicine. In recent years, as breakthroughs have been made in the large-scale production of fullerenes and their derivatives, the application research of fullerenes has also made great progress. Studies have shown that fullerene derivatives have a wide range of anti-tumor activities, such as immune enhancement, anti-oxidation, anti-metastasis, cell cycle arrest, inhibition of tumor angiogenesis and inhibition of multi-drug resistance. Nevertheless, to overcome the limitations of traditional anti-tumor drugs, fullerene derivatives still face some urgent problems to be solved. First, the current research on the anti-cancer mechanism of fullerene nanoparticles is not thorough enough and there is a lack of understanding of the efficient regulation mechanism of tumor proliferation and metastasis. Secondly, it is currently uncertain whether fullerenol will be inside or outside the nucleus. More work needs to be done to determine how nanocarriers reach tumor tissues and drug release to achieve targeted drug delivery and controlled release and how non-toxic nanoparticles themselves can be used directly as anti-tumor drugs before they can be used to treat cancer. Third, the metabolic mechanism and biological safety of fullerene derivatives in the body are still not fully understood. These factors affect the clinical application of fullerene derivatives. At last, fullerene derivatives have unique and interesting size- and shape-dependent optical, electronic and magnetic properties that largely depend on methods to synthesize, purify and characterize them. Therefore, precision syntheses with controlled morphology, size and tightly focused distributions to achieve the targeted functions specifically due to property-determining feature. However, there are no standardized characterization methods of NPs before use in humans that must be a focus for nanomedicine applications. Because the biodistribution and interaction of NPs with proteins are strongly surface- and size-dependent, many NPs will distribute differently and may exhibit undesired effects or even toxicity in a heterogeneous sample. So, it is essential to develop new and improved NPs separation and purification protocols that facilitate the production of optimal nanomedical samples and study the behavior of NPs in vivo.

## Figures and Tables

**Table 1:** Fullerene derivatives with anti-tumor activity.

Fullerene Derivatives	Anti-Tumor Mechanism	In Vitro/In Vivo Model	Main Effects	Ref.
C_60_(OH)_20_	cellular immunity activation	T lymphocytes and macrophages/C57BL/6 mice	improve the immune response to kill tumor cells.	[3]
	tumor metastasis inhibition	EMT-6 breast cancer metastasis model	inhibit tumor cell proliferation	[4]
Gd@C_82_(OH)_22_	cellular immunity activation	dendritic cells and macrophages /C57BL/6	activate Th1 Immune Responses	[5,6]
	angiogenesis suppression	malignant human breast cancer models/cancer stem cells	reduce tumor microvessels density (MVD)	[7,8,9]
	inhibition of oxidative stress	hepatoma cell	normalize the activity of enzymes related to oxidative stress	[10]
	cell cycle regulation	MCF-7 and ECV304 cell	induce the G0/G1 phase arrest	[11,12]
	tumor metastasis inhibition	BALB/c nu/nu female mice	inhibit MMP-2 and MMP-9 with high antitumoral efficacy	[13]
C_60_(OH)_24_	inhibition of oxidative stress	A549 cells	attenuate oxidative stress-induced apoptosis	[14]
Gd@C_82_-(EDA)_8_	maintain a reactive oxygen species (ROS) balance	human epidermal keratinocytes-adult	scavenge hydroxyl radicals	[15]
β-alanine modified gadofullerene nanoparticles (GFNPs)	tumor photodynamic therapy	melanoma cancer cells/female BALB/c nude mice	disrupt tumor vasculatures	[16]
C_60_–Cisplatin nanocomplexs	drug delivery carriers	lewis lung carcinoma cells	enhance the toxic effect of cisplatin on lung cancer cells	[17]
C_60_-Berberiner nanocomplexes		CCRF-CEM cells	inhibit the proliferation of CCRF-CEM cells	[18]

## Data Availability

The data presented in this study are openly available.
